# Nutrition for healthy longevity: the past, the present and the future

**DOI:** 10.1016/j.jnha.2026.100812

**Published:** 2026-02-19

**Authors:** Philipe de Souto Barreto, Jorge G. Ruiz

**Affiliations:** a IHU Health Age, Toulouse, France; b Institut du Vieillissement, Gérontopole of the Centre Hospitalo-Universitaire of Toulouse, Toulouse, France; c CERPOP, UMR 1295, Université de Toulouse, Toulouse, France; d Memorial Healthcare System, Hollywood, Florida and Florida Atlantic University Charles E. Schmidt College of Medicine, Boca Raton, Florida, United States of America

Nutrition is undoubtedly one of the major behavioral drivers of health during aging. It has been recognized as a cornerstone of health since the time of Hippocrates, 400 BCE, in the well-known *On Regimen in Acute Diseases* [1]. Over the past few decades, a substantial and growing body of evidence has shown the importance of healthy diets for preventing and treating chronic diseases [[2], [3], [4]], which tend to accumulate in number and severity with advancing age.

Although the treatment of chronic conditions represents a crucial aspect that informed the organization of healthcare systems around the world and has contributed to the increase in life expectancy, over recent decades, the focus of aging research and care has progressively shifted from the treatment of single diseases to the maximization of functional capacities and their optimal maintenance even in late life [5]. This paradigm shift has been enabled and fostered by the World Health Organization (WHO) which introduced the concept of Healthy Aging in its World Report on Ageing and Health published in 2015 [6]. Indeed, for the WHO, healthy aging is not the absence of disease, but rather the process of developing and maintaining the functional abilities that enables well-being in older age; this is determined by the so-called intrinsic capacity (IC), the environment, and their interaction. IC encompasses all internal physical and mental attributes of an individual and it is commonly operationalized through the domains of locomotion, cognition, vitality, psychology, and sensory function (vision and hearing). In addition, the WHO developed a person- and function-centered healthcare pathway for older individuals, the Integrated Care for Older People (ICOPE) [5], in which IC represents the core element that triggers and guides the different steps in the care pathway.

Within this conceptual framework, nutrition emerges as a central element contributing to healthy longevity. There is a bulk of evidence showing that nutritional aspects, such as healthy dietary patterns and nutrients, contribute to maintaining motor performance [7] and cognitive function [8,9], as well as vitality (as measured by handgrip strength [10]). A more limited but increasing literature also supports that nutrition may play a role in psychology and the sensory domain, in particular, vision. Despite that, there is limited evidence showing the role of nutrition in all these IC domains in the same individuals; this is important because the domains of IC interact with one another to determine healthy aging. A recent article by Tessier et al. [11] using data from more than 100,000 people, followed for up to 30 years, found that several healthy dietary patterns predicted successful aging and its main components, including optimal physical, cognitive and psychological capacity (all of them domains of IC).

The Journal of Nutrition, Health & Aging (JNHA) has made substantial contributions to the fields of nutrition and Healthy Longevity in the past two decades, with a particular emphasis in the past few years [[12], [13], [14], [15], [16], [17]]. With almost 2 million downloads and views in the year 2025, our ambition as Chief Editors of the Journal is to consolidate the JNHA as the journal of choice for high-level publications in the field of Nutrition for Healthy Longevity around the globe. Our interest is to publish cutting-edge studies examining the role of nutrition and its different components in maintaining/maximizing IC during aging, including (but not limited to) dietary patterns, macro and micronutrients, nutritional supplementation, but also nutrition-related surrounding areas, such as the social aspects of cooking and eating. Investigations examining the biological mechanisms through which nutritional elements contribute to healthy longevity are highly welcome, in particular translational studies with immediate or near-term applicability to human health. In this context, linking nutrition to actionable biological aging targets and how they interact to determine IC under a geroscience perspective is highly encouraged. Indeed, the effects of nutritional elements in modulating the main drivers of aging, the so-called hallmarks of aging” [18], constitute a field of major scientific interest, as it opens the avenue for the development of gero-nutraceuticals (nutrition supplementation with a geroprotector action) enabling healthy longevity. Furthermore, works devoted to testing nutritional interventions within the frame of the ICOPE program may also contribute to establishing a pragmatic nutritional approach to healthy longevity.

Therefore, the JNHA will launch a special issue in January 2027 exclusively dedicated to publishing works on Nutrition for Healthy Longevity. In the January 2027 Journal issue, we will also publish the first expert consensus/recommendation on Nutrition for Healthy Longevity, with the participation of experts from several scientific areas, including nutrition, gerontology and geriatrics, each of the IC domains, and geroscience. We invite the scientific community to submit high-quality original research, reviews, and innovative contributions to the Nutrition for Healthy Longevity special issue.

## CRediT authorship contribution statement

PSB has conceived the manuscript, written the first draft and approved the final version for submission; JGR has contributed to the manuscript conception, revised critically the manuscript with important intelectual content and approved the final version for submission.

## Declaration of Generative AI and AI-assisted technologies in the writing process

No use of AI.

## Declaration of competing interest

No competing interest.
